# Immunosuppressive Property of MSCs Mediated by Cell Surface Receptors

**DOI:** 10.3389/fimmu.2020.01076

**Published:** 2020-07-28

**Authors:** Siyu Liu, Fei Liu, You Zhou, Baeku Jin, Qiang Sun, Shu Guo

**Affiliations:** ^1^Department of Plastic Surgery, The First Hospital of China Medical University, Shenyang, China; ^2^Department of Breast Surgery, Shengjing Hospital of China Medical University, Shenyang, China

**Keywords:** mesenchymalstemcells, immunosuppression, cellularsurfacemolecules, adhesionreceptors, chemokinereceptors, co-inhibitorymolecules

## Abstract

In the past decade, mesenchymal stem cells (MSCs) tend to exhibit inherent tropism for refractory inflammatory diseases and engineered MSCs have appeared on the market as therapeutic agents. Recently, engineered MSCs target to cell surface molecules on immune cells has been a new strategy to improve MSC applications. In this review, we discuss the roles of multiple receptors (ICAM-1, Gal-9, PD-L1, TIGIT, CD200, and CXCR4) in the process of MSCs' immunosuppressive properties. Furthermore, we discuss the principles and strategies for developing receptor-regulated MSCs and their mechanisms of action and the challenges of using MSCs as immunosuppressive therapies.

## Introduction

Stem cells therapy is considered as a new therapeutic approach for treating many diseases. Among them, mesenchymal stem cells (MSCs) are one of the primary stem cells, which have been widely used in clinical practice for more than 10 years, and have been proven to be safe and effective (1, 2). Many studies have shown that the immunomodulatory properties of MSCs were developed by suppressing a wide range of innate and adaptive immune cells, including lymphocytes, natural killer cells (NK cells), macrophages, and dendritic cells (DCs). However, the mechanism of MSCs in the modulation of immune cells is still under debate.

Evidence suggested that the immunosuppressive properties of MSCs were mediated by direct or indirect cell-to-cell communication to educate immune cells and ultimately regulate the disease-specific microenvironment (3, 4). Various surface molecules mediate direct cell-to-cell communication, such as chemokine receptors, co-inhibitory molecules, cytokine receptors, and adhesion molecules (3, 5–7). On the other hand, indirect cell-to-cell communication is achieved by extracellular vesicles secreted by MSCs, which included HGF, TSG-6, TGF-β, PGE2, IDO, IL-10, NO, HO-1, or HLA-G (4, 8–14). It is worth noting that some surface molecules also could be secreted to supernate (15, 16). The mechanism of MSCs-mediated immunomodulation remains controversial, which may be the result of multifactorial pathways. Although soluble paracrine factors are essential for MSCs-mediated immunity, more and more studies have shown that direct cell-to-cell communication has played a vital role in the immunoregulatory ability of MSCs (17). Both cell-to-cell contact dependent and independent pathways are essential to the immunosuppression of MSCs; however, it is challenging to explain the immunosuppression of MSCs mediated by direct cell-to-cell communication, paracrine manner seems to develop its immunosuppression effect all the time. Nevertheless, the direct cell-to-cell communication mediated by surface molecules may be helpful to attain the maximum immunosuppression by MSCs (3, 18, 19). Therefore, we focus on the immunomodulatory properties of MSCs mediated by cell surface molecules. Due to the state of cell surface molecules (membrane proteins and secreted proteins), we tend to call the immunosuppression of MSCs mediated by cellular surface molecules “direct cell-to-cell communication.” We introduce and discuss the latest advances in cellular surface molecules of MSCs and how they interact with immune cells and mediate immunosuppression.

## Mesenchymal Stem Cells

MSCs are described as multipotent progenitor cells and were first identified in bone marrow in 1970 (20, 21). Subsequent reports disclosed that MSCs could differentiate into osteoblasts, adipocytes, and chondrocytes when exposed to appropriate conditions (5). Besides, MSCs could be isolated from a variety of mature tissues, including bone marrow, adipose tissues, umbilical cord blood, and placenta (3, 5, 22). Simultaneously, strong regeneration ability, easy extraction, low immunogenicity, and none tumorigenicity make MSCs an ideal source of stem cells for regenerative medicine, transplantation, and treatment of immune-related diseases (23–25). Those advantages interested researchers and grown clinical relevance, a need to establish a non-ambiguous and broadly accepted definition for these cells arose. Thus, in 2006 (21), the minimal criteria for defining MSCs established by the International Society for Cellar Therapy:

1. Adhere to plastic with fibroblast-like morphology during culture;
2. Ability to differentiate into osteoblasts, adipocytes, and chondrocytes *in vitro*;
3. Expression of specific surface markers such as CD73, CD90, Sca-1, and CD105, and non-expression of CD14, CD11b, CD34, and CD45.

However, subsequent studies have found that MSCs from different tissues might express different specific surface markers. In our previous studies, adipose-derived stem cells (ADSCs) expressed CD10, CD13, and CD49d, and in most cases, bone marrow-derived MSCs or muscle-derived MSCs expressed more CD73, CD90, CD105, and CD44 (5, 26, 27). Thus, the concept of markers used to identify MSCs may be suitable for distinguishing MSCs from different tissue sources. In addition to these specific surface markers, MSCs also expressed other functional surface molecules, such as chemokine receptors, cytokine receptors, adhesion molecules, and co-inhibitory molecules. They performed different functions in different environments (28).

## The Immunomodulatory Ability of Stem Cells

The immunomodulatory properties of MSCs distinguish them from other types of stem cells, including hematopoietic cells, embryonic cells, and induced pluripotent stem cells. Furthermore, many preclinical trials have also shown that the use of MSCs has been an effective way to treat refractory inflammatory diseases, such as acute graft-vs.-host disease (GVHD), autoimmune disease, and ulcer (29, 30). Currently, the detailed mechanism of the immunosuppressive properties of MSCs is still unclear. Increasing evidence indicated that MSCs could develop immunosuppressive properties by regulating the phenotypes and activities of innate and adaptive immune cells. In the microenvironment regulated by MSCs, T cells transformed into a tolerant Treg phenotype; macrophages transformed into an immunoregulatory M2-macrophages phenotype; NK cells transformed into non-free functional status, and MSCs could suppress the production of immunoglobulins and increase the production of regulatory B cells (Bregs) (31–34). Cell-to-cell contact dependent and independent ways to develop its' immunosuppression by blocking immune cell differentiation and cell cycle are described as the exact mechanisms (4, 5, 9, 17). When inflammatory mediators stimulated MSCs (e.g., IL-1β, TNF-α, etc.), PGE-2, TSG-6, TGF-β, and HO-1, etc. (4, 8–14) secreted by MSCs into microenvironment could act on immune cells and indirectly exert their immunoregulatory property. In addition to soluble factors, cellular surface molecules are also essential for the immunosuppression of MSCs. We found that direct cell-to-cell communication via Gal-9 and PD-L1 on the surface of ADSCs was more useful to trigger T cell apoptosis than indirect induction (5). Furthermore, cell-to-cell communication is also accompanied by the secretion of anti-inflammation cytokines by MSCs. It seems that direct cell-to-cell communication is a synergic effect that triggers the secretion of soluble factors, and synergistically promotes immune tolerance.

## Clinical Application Status of MSCs

**Figure 1:**
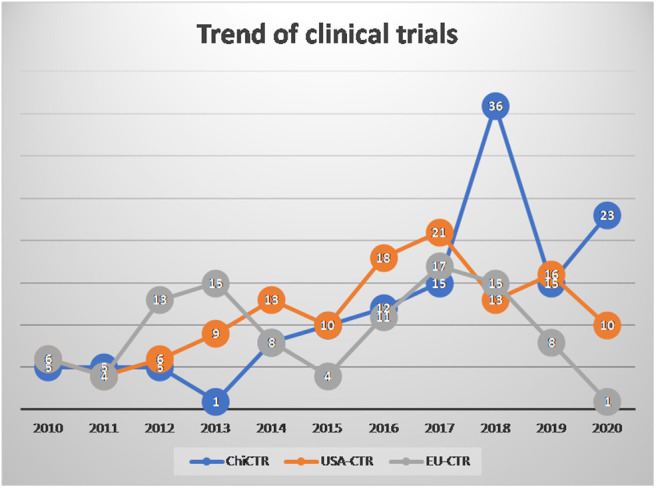
The number of mesenchymal stem cell (MSC) clinical trials registered in 2010-2020.03.30.

From 2010 to 2020, clinical trials registered in the Chinese Clinical Trial Registry (ChiCTR), EU Clinical Trials Registery (EU-CTR), and Americould ClinicalTrials.gov, using the term “mesenchymal stem cells” showed a linear growth trend each year (Figure 1).

Until March 30, 2020, MSCs have been used in a large number of clinical trials worldwide, and the safety of MSCs has been widely verified (29). To our surprise, there were several therapeutic drugs developed on the market based on MSCs (29, 30), but they showed poor results in clinical trials. In our opinion, the poor results are due to the lack of multiple sources and individual differences in MSCs, which in turn leads to repeatability and comparability. Besides, one of the crucial reasons is that the detailed mechanism of action of MSC treatment is still unclear. For example, there are contradictions among direct cell-to-cell communication, MSCs homing, and rapid clearance after intravenous transplantation of MSCs. Those contradictions could be improved by new technologies, such as the immobilization of cell technology (35). Therefore, a detailed study of the mechanism of action of MSCs in the body will be beneficial to the clinical application of MSCs.

In summary, the clinical application of MSCs is still in the exploratory stage. A large number of fundamental researches could reveal the specific mechanism of MSCs treatment under different disease conditions, and then effectively promote the clinical research process using MSCs. Furthermore, the standardization of manufacturing and treatment is also crucial for the clinical application of MSCs. We are optimistic that many stem cell products will meet the criteria for product registration and benefit humankind in the next 5 years.

## Cell Surface Receptor-Mediated MSCs Immunosuppression

Although MSCs have been successfully used as seed cells for tissue repair, there is increasing evidence that MSCs have significant immunosuppressive properties against inflammatory diseases, but its molecular mechanism remains unclear. Extracellular vesicles and other soluble factors secreted from MSCs have been considered as the primary critical anti-inflammatory mediators (36–39), as the intensity of MSCs-EVs is close to the intensity of MSCs. However, some reports disclosed that direct cell-to-cell communication mediated by surface molecules also contributed to the immunosuppression of MSCs (4, 40, 41). Zhou et al. found that the direct cell-to-cell communication had more obvious immunosuppression effect on CD4+ and CD8+ T cells compared to indirect cell-to-cell communication, meanwhile, block some surface receptors would reduce the strength of ADSCs-immunosuppression effect, significantly (5, 10). The different perceptions of confused researchers, and we thought the different environments might cause different results. In the inflammatory environment, the surface receptor may be more critical than the paracrine effect of MSCs, because the inflammatory cytokine enhances the surface receptors expression and promote MSCs immigrate to the target site and exert immunosuppression effect in different stage. Those studies attracted our interests and promoted us to identify specific cell surface molecules expressed on ADSCs (42, 43). These surface molecules, including chemokine and chemotactic receptors, adhesion molecule cytokine and cytokine receptors, co-inhibitory molecules, and other surface markers, have been proven to be related to the immunosuppression properties of tumor cells. However, it is still unknown whether those surface molecules mediate the immunosuppression properties of MSCs. Furthermore, the roles of different surface molecules of MSCs in the immunoregulation of immune cells are still unclear.

In the following, we summarized recent studies describing the interactions between MSCs and different cell populations of the immune system to establish a tolerant microenvironment by direct cell-to-cell communication.

### Adhesion Molecules

Adhesion molecules mediate the first step in cell-to-cell communication between MSCs and target cells expressed on MSCs (ICAM-1, VCAM-1, and P-selecting), which interact with corresponding receptors presented on target cells (6, 44, 45). As mention above, ICAM-1 is the highest expressed adhesion molecules on MSCs, so we focus on ICAM-1 to explain the role of adhesion molecules in the immunosuppression of MSCs.

#### ICAM-1

ICAM-1, also known as CD54, is a transmembrane protein that belongs to the immunoglobulins superfamily. ICAM-1 is mainly expressed in endothelium, leukocytes, fibroblast, and tumor cells (46, 47). Inflammatory cytokines such as IFN-γ, TNF-α, and IL-1β (42, 46, 47) could promote the interaction of ICAM-1 with its ligands LFA-1 or Mac-1, and then mediate the migration of leukocyte to local injury tissues.

Recent studies have shown that ICAM-1 has also been expressed on MSCs and functionally affected MSCs characteristics such as immunosuppression capacity (3, 6, 41, 48). ICAM-1 may play a decisive role in the regulation of immunosuppressive properties. It could act as an adhesion molecule to directly educate immune cells *in vivo* and *in vitro* (6, 41). It is well-known that MSCs could induce polarization of macrophages toward the M2-Mϕ phenotype through ICAM-1/LFA-1 (3, 48). Besides, MSCs were capable of reprogramming microglia into an “M2-like” phenotype (15). In addition to macrophage, ICAM-1 also mediated T cells' proliferation and functional impairment (15, 49). MSCs with higher ICAM-1 expression could inhibit DCs maturation and T cells immune response and even show promising effects in reducing transplantation rejection (45). These studies indicated that direct cell-to-cell communication through ICAM-1 was essential for MSCs to immunomodulate and controlled various immune cells. Moreover, ICAM-1 not only functions through direct cell-to-cell interaction but also promotes the paracrine effect of MSCs, which in turn works with these secreted cytokines synergistically promotes immune tolerance (6, 41, 50). However, different from stimulating protein phosphorylation in the downstream pathway of immune cells, ICAM-1 is considered to play a purely adhesive role in the immunosuppressive effect of MSCs (51). Once the immune cells attached to the inflammatory cytokine-stimulated MSCs, where a high concentration of immunosuppressive effector molecules could act on the immune cells and lead the immune cells to undergo apoptosis, cell cycle arrest, or phenotype-switch. So the Blockade of ICAM-1 could significantly reverse MSC-mediated immunosuppression *in vitro* and *in vivo*, and knockdown INOS or IDO, the immunosuppression effect would eliminate (52, 53), suggesting that adhesion molecules indeed cooperate with the effector molecules.

In conclusion, MSCs with higher ICAM-1 expression may have better immunosuppression ability, which may provide new potential therapeutic applications for some inflammatory diseases, such as asthma, IBD, and diabetic foot. Besides, MSCs with higher ICAM-1 expression could effectively migrate to injury sites and educate more immune cells. The further elucidation of the role of adhesion molecules in immune response will improve our understanding of immunoregulation and help to develop better adhesion molecule targeting therapies.

### Co-inhibitory Molecules

Synergistic co-inhibitory molecules play an essential role in the activation and proliferation of immune cells. At present, PD-1, TIGIT, and Gal-9 are key immune molecules to mediate the immune escape process of tumor cells. In recent years, more and more studies have shown that co-inhibitory molecules on MSCs have also been related to the immunoregulatory ability of MSCs. Therefore, we focus on the Gal-9, TIGIT, and PD-1 families and discuss their immunosuppression properties.

#### Galectins

Galectins are known as a family of the β-galactoside-binding animal lectins expressed on the surface of various cells. Lectin expressed on the cell surface could form lattices and interact with other cells by binding to the corresponding receptor (54). Currently, 11 of the 15 galectins have been identified in human tissues (55). In general, galectins mediate many biological processes, including cell growth regulation, apoptosis, pre-mRNA splicing, intercellular and cell-matrix adhesion, and cell polarity, motility, differentiation, transformation, and signal transduction, as well as the innate/adaptive immunity (56, 57). In the human immune system, galectins play a crucial role in regulating the homeostasis of the microenvironment. Galectin-1 (Gal-1), galectin-3 (Gal-3), and galectin-9 (Gal-9) are the three most exciting representatives of their abilities to mediate immunosuppression. They are perhaps the best understood as an immune checkpoint molecule, and its role in suppressing host immune surveillance is precise, especially Gal-9, as they could induce several kinds of immune cells apoptosis by RAS signaling or calcium-calpain-caspase-1 pathway (58, 59).

##### Gal-9

Gal-9 is a 36 or 39 kDa tandem-repeat galectin with two non-identical carbohydrate recognition domains connected by a linker of variable (60, 61). In recent years, Gal-9 is gradually considered as a checkpoint molecule and as a marker for identifying the therapeutic potency of MSCs (58, 62). The gal-9-mediated immunosuppressive mechanism in MSCs may promote T cells apoptosis or suppress T cells' activities. Once Gal-9 binds to the ligand TIM-3 on the surface of T cells, the downstream pathways NF-κB and AKT will be activated and promote Th cells apoptosis (63, 64). Therefore, the binding of Gal-9 with proper ligands could improve immune tolerance and could be further used in patients with transplant rejection and autoimmune diseases (16, 65, 66). When ADSCs cocultured with activated PBMC, not only Gal-9 but also the secretion of some inflammatory cytokine, such as IL-1β, IL-1α, TNF-α, and IFN-γ were further increased (5). Besides, the increased expression of Gal-9 was associated with STAT and JNK pathways (5, 60, 67, 68). These results indicated that Gal-9 was an anti-inflammatory mediator and could be induced by the inflammatory environment. Inhibiting Gal-9 expression by retrovirus-based approach or blocking the Gal-9/TIM-3 pathway with inhibitors could effectively decrease the immunoregulatory abilities of MSCs (60, 62, 69), indicating that MSCs could target T cells through the surface expression of Gal-9. Thus, many studies have focused on MSCs-mediated antiproliferative effects on T cells. A study by Fan et al. (67) revealed that umbilical cord-derived mesenchymal stem cells (UCB-MSCs) could functionally inhibit the proliferation of CD4^+^ T cells, the differentiation of T cells into Th1 and Th17 cells, and the maturation of DC cells, thereby suppressing the antigen-presenting capacities. Besides, Zhou et al. found that MSCs could inhibit the proliferation of CD4^+^ T cells and CD8^+^ T cells, and even promote apoptosis of both CD4^+^ and CD8^+^ T cells. Furthermore, they also found that MSCs could promote the formation of Treg cells (50). These regulatory effects on T cells may be related to the expression of Gal-9 expressed on MSCs (5, 63, 67). Further animal studies showed similar results from *in-vitro* studies before, under the PBMC co-coculture with recombinant Gal-9, Th1 cells were inhibited while Th2-derived cytokines are predominant (66, 70, 71). We speculate that different expression levels of TIM-3 expressed in Th1 and Th2 cells may lead to the opposite outcome. Gal-9 expressed by MSCs may play a negative role in T cell activity. The mechanism may promote the apoptosis of Th1, Th2, Th17, and CD8^+^ T cells, then promote the formation of Treg cells and eventually lead to a tolerant microenvironment, in which cellular immunity is completely dysfunction, and humoral immunity is a partial disability.

In addition to T cells, TIM-3 was identified on the macrophage. It is well-known that MSCs exert their immunomodulatory effects by promoting polarization of pro-inflammatory macrophages (M1-macrophage) into an anti-inflammatory macrophage (M2-macrophage). Interestingly, Gal-9 also plays a vital role in this process. To our surprise, no studies have been conducted to investigate whether Gal-9 expressed by MSCs mediates macrophage reprogramming. In addition to *in vitro* results, the results of in LPS-induced preeclampsia-like Rats model also indicated that Gal-9 exerted a positive effect on the M2-macrophage polarization (72, 73). Interestingly, once some macrophages reprogrammed into M2-macrophages, the exosomes secreted by these reprogrammed M2-macrophages would synergistically accelerate the reprogramming process of the remaining macrophages into M2 subtype (74). Macrophages are the bridge between the innate and adaptive immune systems. Once macrophages are switched into M2-macrophages, the capacities of the innate and adaptive immune systems will also be affected directly and indirectly. Currently, it is unknown whether Gal-9 on MSCs mediates the polarization of macrophages. Further studies should be conducted to clarify whether Gal-9 on MSCs could mediate M2-macrophage reprogramming. It is worth noting that Gal-9 plays a pivotal role in M2-macrophage polarization, which may reveal the mechanism of immunosuppression of MSCs. When a large number of macrophages are reprogramming into M2-macrophages, the number of antigen-presenting cells (APCs) and macrophages as a clearance function will be insufficient, resulting in the dysfunction of innate and adaptive immune systems. Subsequently, Th and CLT cells will not normally differentiate due to the lack of antigen stimulation. Besides, M2-macrophages will secrete some anti-inflammatory cytokines, such as IL-10, IL-4, and TGF-β, which will turn T and B cells into a regulative phenotype. Therefore, the key mechanism of immunosuppression of MSCs in the inflammatory microenvironment is the Gal-9-mediated macrophage reprogramming (71).

Like Gal-1 and Gal-3, Gal-9 is also secreted into the supernatant (16, 75–77). Although the amount of Gal-9 in the supernatant was negatively associated with the strength of GVHD, TIM-3^+^ T cells do not correlate with transplantation rejection (16). Therefore, the formation of Treg cells may not be caused by Gal-9 on MSCs, but the polarized macrophages regulated by MSCs most likely cause it.

##### Gal-1 and Gal-3

Unlike Gal-9, the immunomodulatory properties of Gal-1 on MSC are still under debate. Although Gal-1 on the surface of MSCs could trigger apoptosis of activated T cells *in vitro*, there is still a lack of *in vivo* studies to show that Gal-1 deficiency on MSCs could downregulate the immunosuppressive capacities of MSCs (78, 79). Gal-1 may not be the essential part of directly mediating the immunosuppressive properties of MSCs. However, it may still be involved in regulating the other functions of MSCs, such as motility and differentiation. A study by Yun et al. revealed that Gal-1 could promote MSCs' motility by modulating NF-κB and Smad2/3 pathways (80). Other studies have also shown that Gal-1 on the surface of MSCs could promote angiogenesis and tumor progression (81–83). The biological activities of Gal-1 could promote not only repair and regeneration but also tumor metastasis in the tumor microenvironment (84, 85). Besides, Gal-3 has been reported to have similar functions (86–88). However, compared to Gal-1, Gal-3 is more similar to Gal-9 in immunosuppression properties. A study by Liu et al. showed that Gal-3 knockdown significantly abolished the inhibitory effect of MSCs on activated peripheral blood mononuclear cells (PBMCs) (77). Moreover, the Gal-3 secreted by UCB-MSCs showed a positive effect on the infarcted myocardium (89). Unlike Gal-9, the expression of Gal-1 and Gal-3 in equine bone marrow-derived stem cells (BM-MSCs) is impaired under inflammatory conditions (90). Galectins play different roles in the systematic immune system and local microenvironment. Galectins, especially Gal-1 and Gal-3 instead of Gal-9, have shown strong immunomodulatory effects *in vitro*, but opposite results *in vivo*. Future clinical studies should focus on explaining why galectins have such different effects *in vivo* and *in vitro* and the development of MSCs in the treatment of inflammatory diseases.

#### Programmed Death-Ligand 1 (PD-L1)

The binding of co-stimulatory molecules such as the ligand of the B7 family CTLA-4/B7-1 to receptors of the CD28 family such as CD28/CD86 is vital for controlling inflammation. Programmed cell death protein 1 (PD-1), also known as CD279, belongs to the CD28/B7 superfamily. PD-1 is mainly expressed on T cells, NK cells, macrophages, DCs, and B cells. PD-L1, also known as B7-H1 (CD274), and PD-L2, also known as B7-DC or CD273, are both ligands of PD-1. PD-L1 is expressed both on non-hematopoietic stem cells and specific subpopulations of hematopoietic stem cells (91–93). In contrast, PD-L2 is expressed primarily inactivated antigen-presenting cells (APCs), such as DCs and macrophages. Still, it could be induced on the surface or cytoplasm of tumor cells and other immunoregulatory cells such as MSCs (31, 94). PD-1 could not only bind to PD-L1 and PD-L2 but also bind to CD80 and RGMb (94). Similar to the CTLA-4/B7 model, PD-L1 generally has a higher expression level than PD-L2 (95). The binding affinity of PD-L1 for PD-1 and PD-L2 for PD-1 are similar (Kd values; 10 nM), but it is important to note that PD-L1 but not PD-L2 had a delayed interaction reminiscent. The striking differences were observed at the level of the association and dissociation characteristics, and the expression level of PD-L1 is significantly higher than PD-L2 on a wide range of human and mouse cells, so PD-L1 is the main negative immune-checkpoint in the programmed cells death protein family (94–96).

Targeting the PD-1/PD-L1 pathway plays a vital role in T cells' homeostasis, and molecular therapeutic drugs such as Nivolumab and Pembrolizumab have been widely used clinically to target PD-1 and PD-L1 and showed promising effects in PD-1^+^ or PD-L1^+^ carcinoma (97–99). In the tumor microenvironment, once PD-1/PD-L1 interacts, T cells would receive negative signals transmitted from PD-L1 expressed on the surface of tumor cells, thereby promote the development of Treg cells and eventually develop the characteristics of tumor immune escape (100).

When PD-L1 was found to be highly expressed on MSCs (101), the role of PD-L1 attracted much attention. Studies have also found that PD-1 expressed on MSCs is essential for maintaining MSCs stem cell properties (102). Furthermore, it is interesting that PD-L1 and PD-L2 were found to be secreted into the supernatant by some type of MSCs, such as human BM-MSCs and Tonsil-derived MSCs (5, 91). Many studies that focused on the immunosuppressive mechanism of MSCs mediated by the PD-1/PD-L1 pathway have obtained similar results to the conclusion in the field of tumors.

Targeting PD-1/PD-L1 is of great significance for the immunosuppressive properties of MSCs on T cells. When the PD-L1 signaling on MSC was blocked, the immunosuppressive capacities of MSCs were significantly eliminated (5, 103). A series of studies on the PD-1/PD-L1 interaction between MSCs and T cells showed that MSCs had different regulatory effects on different phenotypes of T cells. The secretion of inflammatory cytokine IL-17 by Th17 is suppressed by MSCs-mediated PD-1/PD-L1 interaction (40, 104). Interestingly, there was also found that MSCs inhibited the differentiation of T cells into Th17 cells but did not affect the production of IL-17 by mature Th17 cells (40). Although the mechanism is still unknown, we hypothesize that the mature Th17 will reduce PD-L1 expression. Both Th1 and Th2 cells are essential to the immune system; Th1 cells are involved in cellular immunity, while Th2 cells are responsible for humoral immunity. MSCs could inhibit the Th1 cells while promoting Th2 cell polarization (105). However, both Th1 and Th2 cells seemed to be induced toward the apoptotic pathways in different degrees (6). After MSCs educated t cells, the quality and quantity of Treg cells increased significantly, which may be caused by direct cell-to-cell communication between T cells with MSCs or the secretion of IL-10 and TGF-β by MSCs (31, 106–108). Some studies have shown that the PD-1/PD-L1 interaction only promoted T cells apoptosis but was not related to the elevated levels of immunosuppressive cytokines, such as TGF-β and IL-10. In other words, PD-1/PD-L1 interaction does not promote the formation of Treg cells. Different research results require more experimental confirmation. PD-L1 expressed on MSCs is not stably expressed. It has been shown that inflammatory cytokines such as IFN-γ and TNF-α were associated with the elevated level of PD-L1 on MSCs and then affected the immunosuppressive properties of MSCs (91). Besides, other soluble factors such as granulocyte-macrophage colony-stimulating factor (GM-CSF) and polyinosinic-polycytidylic acid could also increase the PD-L1 expression (100, 104). In conclusion, in an inflammatory microenvironment, pro-inflammatory factors could increase the expression of PD-L1 on the surface of MSCs, eventually, lead irreversible hypoergia and cell death, and promote the formation of Treg cells with more immunosuppressive capacities (91, 109, 110). However, knocking-down the expression of PD-L1 on MSCs or blocking the PD-1/PD-L1 signaling pathway will result in the loss of the immunosuppressive function of T cells (5, 111).

In addition to T cells, the PD-1/PD-L1-mediated signaling pathway is also involved in the regulation of other types of immune cells. Studies have shown that MSCs could inhibit the activation, proliferation, and immune function of microglial cells (111) and suppress the proliferation, differentiation, migration, and immunoglobulin-secreting properties of B cells (34).

As mentioned above, the PD-1/PD-L1 interaction is essential for controlling T cells homeostasis between MSCs and T cells. Still, few results have focused on other immune cells, especially APCs (e.g., DCs) and macrophages. It is well-known that MSCs regulate DCs differentiation and macrophages polarization, and the PD-1/PD-L1 signaling pathway mediates the immunosuppress capability of APCs. Due to the crucial role of APCs in the innate and adaptive systems and in regulating immune cell differentiation, the role of APCs in inflammatory diseases is more important than adaptive immune cells. More attention should be paid to the immunosuppressive capacities of APCs mediated by PD-1/PD-L1 in the future.

#### T-Cell Immunoglobulin and ITIM Domain (TIGIT)

TIGIT, also known as Vstm3, VSig9, or WUCAM, is a newly identified co-inhibitory receptor by Yu et al. (112). TIGIT was found to be expressed in a variety of immune cells, including NK cells, memory T cells, M2-macrophages (M2), Treg cells, tumor-infiltrating lymphocytes (TILs), and individual immunoregulatory cells (e.g., tumor cells and MSCs) (113–119). In the tumor microenvironment, the highly expressed TIGIT in TILs may cause local immune tolerance and promote cancer metastasis and progression by modulating the activation of NK cells, DCs, and T cells.

**Figure 2:**
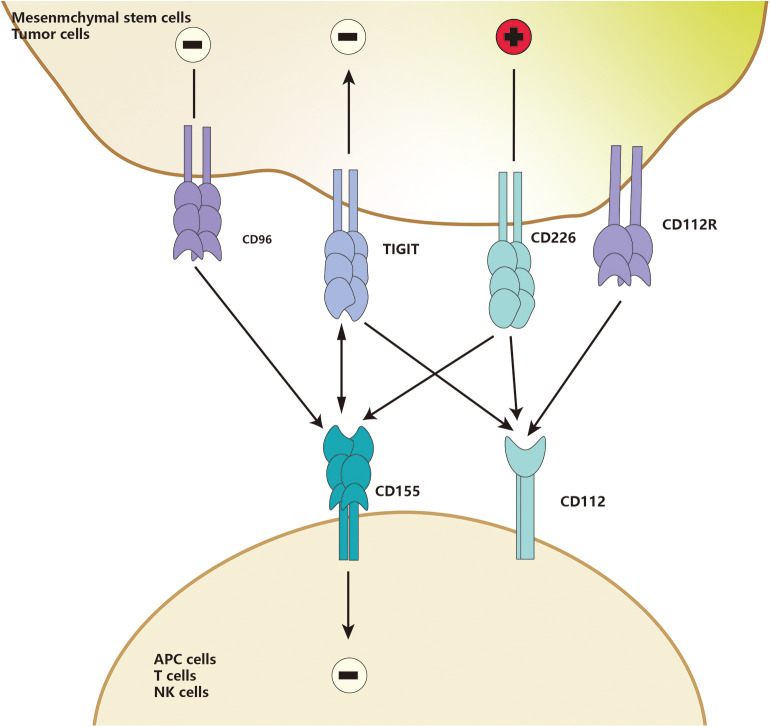
TIGIT and CD226 are detected in MSCs. CD155 and CD112 are detected in APCs. Upon binding, induce co-costimulatory or co-inhibitory signals for CD226 and TIGIT, respectively, CD112 could also deliver an inhibitory signal when engaged by TIGIT.

TIGIT is part of a ligand/receptor network, and the structure of the network is complicated. In humans, two TIGIT interact with two CD155 to form a unique tetramer structure, known as the “lock-and-key interaction” by Stengel et al. (120). Herein, TIGIT could be replaced by CD226, and CD112 could replace CD155. Besides, CD96 or CD112R may also compete with those ligands (121). In the “lock-key” structure, TIGIT could compete with CD226, a co-stimulatory molecule of this network, to bind to high-affinity receptors such as CD155 and PVR, or weakly interact with CD112 (120, 122) (Figure 2), or even block the interaction between CD226 and CD155 in a dose-dependent manner (123–125). Because TIGIT could not only bind to PVR with about 100-fold higher affinity than CD226 but also disrupt the homodimerization of CD226, thus eventually block the interaction of CD226 and its ligands (112, 126). Furthermore, both PVR and CD112 have bidirectional roles in immune activation and inhibition, which is similar to the CD28/CTLA-4-CD80/CD86 family.

As an essential immune checkpoint, TIGIT is generally considered as a receptor to mediate intracellular signaling transduction. It was reported that the ligand CD155, also called PVR, had an ITIM motif at the cytoplasmic tail, which could be phosphorylated and trigger activation of downstream wnt/β-catenin pathway (112, 127). TIGIT/PVR seems to be a bidirectional pathway, which is widely involved in immunosuppressive responses of different immune cells, such as monocytes, macrophages, DCs, T cells, and NK cells (112, 124, 128). CD155 was found to be mainly expressed on APCs (112, 119, 129, 130). When macrophages are exposed to LPS or specific pro-inflammatory cytokines, the expression of PVR on macrophages increases significantly. When TIGIT-FC binds to PVR, macrophages polarize to be tolerant M2-macrophages to increase the anti-inflammatory cytokines such as IL-10 and TGF-β and decrease the pro-inflammatory cytokines such as IL-1β and TNF-α (129). A study by Noguchi et al. (119) showed that overexpression of TIGIT in porcine aortic endothelial cells (PAOECs) suppressed M1-macrophage polarization. Interestingly, TIGIT was found to be expressed only on M2-macrophages but not M1 macrophage, suggesting that TIGIT expression promotes M2-polarization (119). Surface TIGIT may likely bind to PVR on other cells and eventually affect other immune cells, such as T cells and NK cells. Meanwhile, the complex inhibitory effects of TIGIT on T cells has attracted widespread attention. TIGIT directly suppresses T cells proliferation and switches them into a tolerant phenotype, called regulatory T cells. These regulatory T cells are then activated to inhibit the secretion of pro-inflammatory cytokine and increase secretion of anti-inflammatory cytokines, eventually blocking the co-stimulatory signal of CD226 through the mechanism (Figure 2) mentioned above. The immunosuppressive properties of TIGIT are close to the TIGIT-educated APCs phenotypes, such as macrophages and DCs (112, 123, 124, 130).

In last year, we found that TIGIT could also be expressed on ADSCs. Although TIGIT has recently become a new immune checkpoint for cancer immunity (113, 121, 131), few studies have reported on TIGIT expressed by MSCs. CD226 and its ligand CD155 have been reported to be associated with impaired NK cells and T Cells function, while MSCs have the same functions. Moreover, MSCs also express CD226 and TIGIT. So does TIGIT mediate the immunosuppression of MSCs on NK and T cells? More experiments are needed. In some special conditions, such as platelet lysate, the decreased expression of PVR and Nectin-2 in MSCs will weaken the inhibitory capacities of MSCs to T cells and NK cells, decrease PGE2 secretion, and increase IL-6 and IL-8 secretion (33, 132). Besides, NK cells showed a significant decrease in DNAM-1 expression when co-cultured with BM-MSC or hADSCs (133). It is unclear whether TIGIT mediates the immunosuppressive effects of MSCs.

### Chemokine Receptor

#### CXCR4 (CD184)

CXCR4, also known as CD184, is one of the most crucial chemokine receptors and the main factor influencing MSCs homing, in which these were circulating MSCs migrated to the target tissues and interacted with the ligand stromal cell-derived factor-1 (SDF-1) (134). Under the interaction with SDF-1, CXCR4^+^ cells will migrate to the target site according to the gradient of SDF-1.

It is well-known that the up-regulation of CXCR4 may enhance MSCs' migration. Under the induction of SDF-1, CXCR4^+^ MSCs could migrate to the target site for repair. Similarly, the expression level of CXCR4 on MSCs is related to the local microenvironment. SDF-1 pretreatment could promote the expression of CXCR4 in MSCs (135). Hypoxia conditions or other oxidative stress conditions have the same effect as SDF-1 pretreatment (136–138). Since the inflammatory response promotes the secretion of SDF-1, MSCs may express more CXCR4 when the human body is in an inflammatory state. Besides, MSCs with higher CXCR4 expression were considered to have more robust tissue-regeneration capacities, and this could be proved by transfecting CXCR4 into MSCs or using other methods further to increase CXCR4 expression levels (139, 140). Furthermore, CXCR4 transfection did not affect the biological characteristics and vitality of BM-MSCs (141) but enhanced the targeted recruitment and survival of transplanted MSCs (142). Is the establishment of MSCs overexpressing CXCR4 a great way to improve clinical stem cell transplantation, especially autologous fat transplantation? Focusing on the immunoregulation of MSCs, CXCR4 also plays a vital role in inflammatory diseases. Nan et al. revealed that CXCR4 overexpression might reduce colon inflammation in rats while co-transfecting with IL-35 (143). On the one hand, MSCs with high-level CXCR4 have more robust immunomodulatory capacities by reducing the number of DCs and Th17 cells and increasing the number of M2-macrophages and Treg cells (143). On the other hand, CXCR4 deficiency suppresses the immunomodulatory and homing abilities of MSCs (144). These lead to the downregulation of inflammatory cytokines IL-6 and TNF-α and up-regulation of anti-inflammatory IL-10 (140, 143).

Although CXCR4 has been proved to play a crucial role in the regenerative capacities of MSCs, it is unclear how CXCR4 educates the immune cells phenotype switch (145). Which role does directly with immune cells play in this process?

#### CD200

CD200, a member of the transmembrane surface immunoglobulin superfamily, expressed on various types of cells, such as T cells, B cells, vascular endothelial cells, and APCs as well as MSCs (28, 101, 146, 147). Besides, the ligand CD200R is expressed on myeloid cells, particularly monocytes and macrophages.

Interestingly, the level of CD200 on MSCs may increase due to the inflammatory environment or hypoxic conditions (148, 149). Bruno Delorme discovered that CD200 could be expressed on cultured and expanded BM-MSCs, and realized that CD200 expression might be related to the immunoregulatory properties of MSCs (150). Recent studies have further shown that CD200 has been associated with reducing infiltration of inflammatory cells, expansion frequency of myeloid progenitor cells, and improving inflammation (149, 151). Another mechanism may be related to promoting the release of anti-inflammatory factors, such as TGF-β and IL-4. Thus, inhibiting CD200 expression by shRNA or by antagonist-mediated neutralization is effectively to suppress the immunoregulatory properties of MSCs (152, 153). However, immunosuppression properties depend on the CD200/CD200R pathway and are also related to macrophages. Once educated by CD200^+^ MSCs, macrophages would lose their pro-inflammatory properties and begin to polarize into tolerant M2-macrophages (4, 154). Whether CD200/CD200R mediates other immunosuppressive effects on other immune cells is still unknown.

In general, the CD200/CD200R pathway not only promotes the osteogenesis and immunosuppressive properties of MSCs and the secretion of anti-inflammatory cytokines by MSCs but also educates the pro-inflammatory immune cells to polarize into an anti-inflammatory phenotype.

## Perspective

To date, these refractory inflammatory diseases have plagued researchers for many years. MSCs are one of the most crucial seed cells in regenerative medicine and have strong immunoregulatory abilities. So far, there are two main pathways to achieve immunosuppressive properties of MSCs, namely paracrine soluble factors, and receptor-mediated direct cell-to-cell communication. Soluble factors include HGF, TGF-β, PGE2, IDO, IL-10, NO, HO-1, HLA-G and exosomes, and exosomes are considered to be the foremost effective means to achieve immunosuppressive properties of MSCs. However, we believe that direct cell-to-cell communication is essential for the immunosuppressive properties of MSCs. In preclinical studies, the successful promotion of MSCs-mediated immune cells phenotype switch has excited all investigators in the field. Therefore, the use of MSCs mediated by multiple cell surface receptors has the potential to overcome certain refractory immune diseases, such as GVHD, diabetic ulcer, and autoimmune diseases. Still, most researches remain in the fundamental stage, and there are a few animal experiments (Table 1). However, the application of MSCs in clinical treatment must be careful; the safety and effectiveness of these MSCs in clinical treatment must be ensured. Before the clinical usage of MSCs, it is necessary to understand the underlying mechanisms of action and the immunosuppressive properties of MSCs.

**Table 1:** Cell surface receptor expressed on mesenchymal stem cells that in the inflammatory microenvironment, which has been reported to have the potential to treat refractory disease by regulating the activity of different immune cells.

**Receptor type**	**Ligand type**	**Target cells**	**Disease or microenvironment**	**References**
ICAM-1	LFA-1	Macrophage T cells Microglia DC cells Neutrophils	T cell Ag receptor' activated CD3+ pan-T cells activation	(41)
			Autoimmune thyroiditis	(6)
			Bony defect	(32)
			Improve cerebral infarction	(3, 48)
			Alzheimer's disease	(15)
			Multiple sclerosis	(49)
			Ischemic stroke	(50)
Galectin-9 (sGal-9)	TIM-3	T cells DC cells Macrophage	αCD28/OKT3- activated PBMC	(60)
			Hepatocyte chronic infection caused by HCV	(63)
			Chronic HCV infection and promote immunotolerance	(64)
			GVHD	(16, 45, 65)
			Autoimmune diabetes in NOD mice	(66)
			Autoimmune cholangitis	(67)
			Multiple sclerosis	(68)
			PHA activated PBMC	(69)
			Normal PBMC or abortion-PBMC	(70)
			LPS-Induced Preeclampsia-Like Impairment	(72)
			LPS-induced M1-macrophage	(73)
			Experimental hypersensitivity pneumonitis	(71)
Galectin-1 sGal-1		T cells	PHA-stimulated PBMC	(76)
			Ovalbumin-induced DTH model	(76)
			Free-serum in α-MEM essential medium	(80)
			Mice injected orthotopically with breast carcinoma cells or subcutaneously with melanoma cells	(81, 83)
Galectin-3 sGal-3		T cells	Induced medium which contained L-DMEM, 5% FBS, VEGF (10 ng/mL), and bFGF (2 ng/mL)	(86)
			Direct coculture system	(87)
			Complete medium supplemented with 10% FBS	(88)
			Complete medium	(90)
			Complete medium supplemented with 11% FBS	(77)
PD-L1 sPD-L1	PD-1 CD80 RGMb	T cells microgila	Anti-CD2/CD3/CD28 microbeads stimulated CD3+ T cells	(91)
			Autoimmune diabetes mice model	(92)
			Staphylococcal enterotoxin B/ Dynabeads/ anti-CD3 / anti-CD28 activated PBMC	(103)
			Anti-mouse CD3/CD28 Dynabead activated T cells	(40)
			Anti-mouse CD3/CD28 Dynabead activated PBMC	(105)
			Anti-CD2/CD3/CD28 microbeads stimulated CD3+ T cells	(91)
			Multiple myeloma mice model	(107)
			Murine Collagen-Induced Arthritis mice model	(108)
			Anti-CD3/CD28 activated CD4+ CD25- T cells	(109)
			Anti-CD3/CD28 activated Treg cells	(110)
			LPS activated microglia	(111)
			CpG ODN, rCD40L, anti-Ig, IL-2, and IL-4 treated B cells	(34)
PD-L2	PD-1 CD80 RGMb	T cells microglia	PHA activated CD3+ T cells	(31)
			Anti-CD2/CD3/CD28 microbeads stimulated CD3+ T cells	(91)
CXCR4	SDF-1		Experimental colitis mouse model	(134)
			Traumatic Brain Injury rats model	(135)
			The I/R injured liver mouse model	(7, 137)
			Mouse model	(139, 155)
			Cartilage defect Rabbitsmodel	(156)
			Experimental mouse models	(141)
			A rat model of acute myocardial infarction.	(142)
			Mice model	(157)
			Inflammatory bowel disease mouse model	(143)
			Chemoinvasion assay	(144)
			A mouse model of DR	(140)
CD200	CD200R	Macrophage Monocyte	A myocardial infarction (MI) model of SD rats	(145)
			Mice model	(146, 148)
			Co-culture with PBMC condition	(158)
			Corneal injury mice model	(149)
			hMSCs co-culture with human primary hepatocyte	(151)
			The acute phase of the stroke rat model	(153)
			Ischemic brain injury rat model	(152)
			THP-1 macrophage co-culture with MSCs	(154)
			Abortion mouse models	(4)

*Cellular surface receptors expressed on MSCs, which have been or have the potential to be used in immunosuppression of stem cell therapy and their respective targeting agents and target cells in the specialized microenvironment (due to the inadequate number of researches on the role of TIGIT in the immunosuppression of MSCs, we have not listed TIGIT related studies)*.

**Figure 3:**
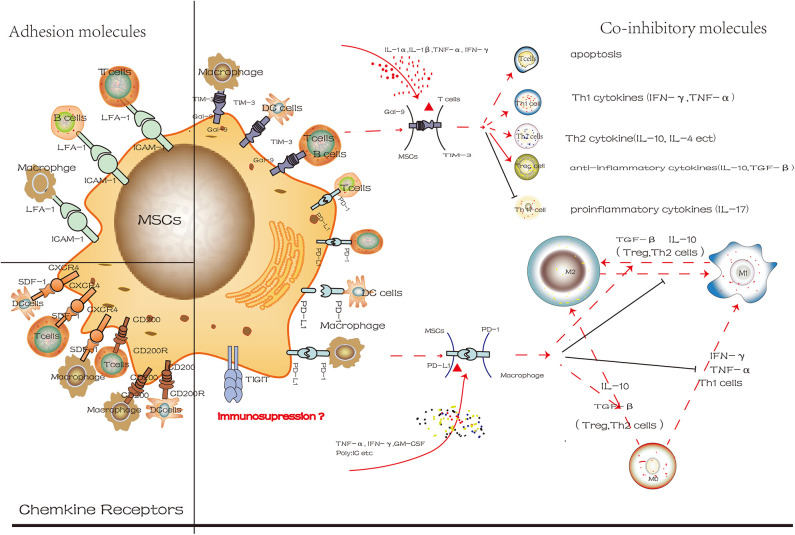
The different degree of cell surface molecules on MSCs in the inflammatory microenvironment is attractive targets for cellular surface receptor targeting therapies. These molecules mainly include co-inhibitory molecules, chemokine receptors and adhesion molecules. And we use Gal-9/TIM-3 and PD-L1/PD-1 to exhibit its mechanism in control of immune cells phenotypic change (red line represents positive effect while the black line represents the negative effect).

Surface molecules contribute to the immunosuppressive properties of MSCs and regulate the phenotype and activity of immune cells (Figure 3). Different surface molecules contribute to the immunosuppressive properties of MSCs in different stages indicated that similar to CXCR4, ICAM-1 facilitated the migration of MSCs to target cells at the site of injury. Subsequently, more MSCs will pool together, and then the co-inhibitory molecules on the MSCs will interact with immune cells directly, eventually regulate the microenvironment. These results indicated that transforming MSCs to a special status by altering the expression of surface molecules on MSCs might be beneficial for immunoregulatory properties. However, so far, most studies have used immune cells and MSCs co-culture system *in vitro*. Still, this method could not reflect the actual development of microenvironment and is limited in practical clinical applications. However, how to transform MSCs to express more molecules that are conductive to immunosuppression, and to maintain the capacities of stem cells, the safety and effectiveness of clinical application are the main issues we need to face in the future.

## Author Contributions

SL, SG, YZ, and QS designed this review. SL and FL collected literatures and conducted the analysis of data. SL and SG drafted and revised the manuscript. FL and BJ wrote the manuscript. All authors contributed to review the manuscript, read, and approved the final manuscript.

## Conflict of Interest

The authors declare that the research was conducted in the absence of any commercial or financial relationships that could be construed as a potential conflict of interest.
